# Centromedian Nucleus of the Thalamus Deep Brain Stimulation for Genetic Generalized Epilepsy: A Case Report and Review of Literature

**DOI:** 10.3389/fnhum.2022.858413

**Published:** 2022-05-20

**Authors:** Shruti Agashe, David Burkholder, Keith Starnes, Jamie J. Van Gompel, Brian N. Lundstrom, Gregory A. Worrell, Nicholas M. Gregg

**Affiliations:** ^1^Department of Neurology, Mayo Clinic, Rochester, MN, United States; ^2^Department of Neurosurgery, Mayo Clinic, Rochester, MN, United States

**Keywords:** deep brain stimulation, centromedian nucleus of the thalamus, idiopathic generalized epilepsy, genetic generalized epilepsy, neuromodulation centromedian thalamic stimulation for generalized epilepsy

## Abstract

There is a paucity of treatment options for cognitively normal individuals with drug resistant genetic generalized epilepsy (GGE). Centromedian nucleus of the thalamus (CM) deep brain stimulation (DBS) may be a viable treatment for GGE. Here, we present the case of a 27-year-old cognitively normal woman with drug resistant GGE, with childhood onset. Seizure semiology are absence seizures and generalized onset tonic clonic (GTC) seizures. At baseline she had 4–8 GTC seizures per month and weekly absence seizures despite three antiseizure medications and vagus nerve stimulation. A multidisciplinary committee recommended off-label use of CM DBS in this patient. Over 12-months of CM DBS she had two GTC seizure days, which were in the setting of medication withdrawal and illness, and no GTC seizures in the last 6 months. There was no significant change in the burden of absence seizures. Presently, just two studies clearly document CM DBS in cognitively normal individuals with GGE or idiopathic generalized epilepsy (IGE) [in contrast to studies of cognitively impaired individuals with developmental and epileptic encephalopathies (DEE)]. Our results suggest that CM DBS can be an effective treatment for cognitively normal individuals with GGE and underscore the need for prospective studies of CM DBS.

## Introduction

Genetic generalized epilepsies (GGEs) consist of epilepsies with generalized onset seizures and generalized spike wave discharges based on the 2017 ILAE classification (Scheffer et al., 2017). Idiopathic generalized epilepsy (IGE) is a subset of GGEs and is comprised of childhood absence epilepsy, juvenile absence epilepsy, juvenile myoclonic epilepsy, and epilepsy with generalized tonic clonic (GTC) seizures alone (Scheffer et al., 2017). Drug resistant GGE, seen in 12–40% of individuals with GGE (Colleran et al., 2017; Gesche et al., 2020) is an undertreated group that lacks any approved surgical or neuromodulation therapies.

Anterior nucleus of the thalamus (ANT) deep brain stimulation (DBS) has a CE (Conformitè Europëenne) mark and FDA (Food and Drug Administration) approval for adjunctive therapy for adults with drug resistant focal (partial) onset seizures (Fisher, 2013; Li and Cook, 2018). Some evidence suggests that ANT DBS is particularly effective for seizure networks that involve the limbic system, supporting a network theory of DBS for epilepsy (Salanova et al., 2021). Beyond ANT DBS, additional subcortical targets continue to be explored in specific types of epilepsies (Wille et al., 2011; Li and Cook, 2018). The centromedian (center médian) nucleus of the thalamus (CM) is one of the intralaminar thalamic nuclei, and along with the parafascicular (Pf) nucleus constitutes the caudal group of the intralaminar nuclei (Jones, 2012). The intralaminar nuclear group, including the CM nucleus, has diffuse cortical projections, and functions as an extension of the brainstem ascending reticular activating system (Jones, 2012). The CM nucleus has been implicated in attention and sleep-wake regulation (Velasco et al., 1987, 2021; Fisher et al., 1992; Velasco M. et al., 2001; Yan et al., 2018; Kokkinos et al., 2020; Alcala-Zermeno et al., 2021). The CM and Pf nuclei additionally have significant basal ganglia projections to sensorimotor, and associational and limbic striatal regions, respectively (Jones, 2012).

Thalamocortical connectivity is altered in GGE both structurally and functionally, and there is activation of CM during generalized spike wave discharges seen in GGE (Tyvaert et al., 2009; O’Muircheartaigh et al., 2012). Modulation of this altered network connectivity may be achieved via CM DBS (Velasco et al., 2006, 2021; Jones, 2012; Fisher, 2013). By interacting with diffuse cortical areas both directly, and indirectly via subcortical structures, CM DBS may alter the hypersynchrony of this aberrant loop (Tyvaert et al., 2009; O’Muircheartaigh et al., 2012; Fisher, 2013; Ilyas et al., 2019). The current evidence for CM DBS is based on relatively small and heterogenous patient cohorts, which include individuals with developmental and epileptic encephalopathies (DEE), and generalized, poorly localized, and focal onset seizures (Velasco et al., 1987, 1993, 2021; Velasco F. et al., 2001; Velasco M. et al., 2001; Velasco et al., 2002; Valentín et al., 2013; Yan et al., 2018; Alcala-Zermeno et al., 2021). The most substantial evidence for CM DBS exists for individuals with Lennox-Gastaut syndrome (LGS), with a recent prospective open-label trial of 20 patients with LGS reporting a 90% responder rate >50% reduction in seizures) (Cukiert et al., 2020). A recent prospective double-blind randomized trial of CM DBS for LGS had a non-significant difference in clinical responder rate (as per patient seizure diary) between the active and control stimulation groups (*p* = 0.25), but a significant reduction in electrographic seizures as recorded by 24 h ambulatory EEG (*p* = 0.05) (Dalic et al., 2022). To date, we are aware of 2 published reports of CM DBS for patients with GGE and normal cognition, including a total of 6 patients (Valentín et al., 2013; Cukiert et al., 2020). There are two additional case reports of patients treated with a responsive neurostimulation device targeting the CM nucleus (Kokkinos et al., 2020; Welch et al., 2021). All subjects were reported to be responders (>50% reduction in seizure frequency).

This report discusses the case of a 27-year-old cognitively normal woman with GGE and childhood onset absence and GTC seizures who was treated with CM DBS. She had a significant reduction in GTC seizures with CM DBS, which was maintained at her 12-month post implant visit. This work encourages future prospective studies of CM DBS for cognitively normal individuals with drug resistant GGE.

## Case Report

A 27 year old right handed woman with seizures starting at age 5 presented to our center for comprehensive evaluation. Her seizures were mainly of three types. Absence seizures began at 5 years of age were not controlled by medications, which continued to occur during adulthood either daily or weekly with variable frequency. Later she had onset of GTCs, which were without any clear focal features. The third type of seizure began in middle school during which she would wander with intermittent twitching of her hands and lips (suspected to be absence status epilepticus). At the time of presentation to our facility she reported that the frequency of her GTCs was 1–2 per week in the preceding 6 months. She had multiple hospitalizations with status epilepticus or following injury sustained during GTC seizures. Episodes of blank staring remained variable from daily to weekly, and it had been years since her last episode of absence status epilepticus with prolonged confusion and wandering. Recently she had reported two events with staring and raising of the left arm, which were found to be psychogenic non-epileptic seizures (PNES) on outside EEG monitoring.

Her history was significant for premature birth at 24 weeks and meningitis at 6 months of age. She suffered from anxiety, depression, and had a history of a suicide attempt. Past antiseizure medications (ASMs) include valproate, ethosuximide, levetiracetam, and topiramate, which were discontinued due to lack of efficacy or side effects (mood and cognitive effects). At the time of presentation, she was taking lamotrigine 250 mg twice daily, zonisamide 400 mg twice daily and oxcarbazepine 150 mg twice daily, and vagus nerve stimulation therapy which had modest benefit.

**FIGURE 1:**
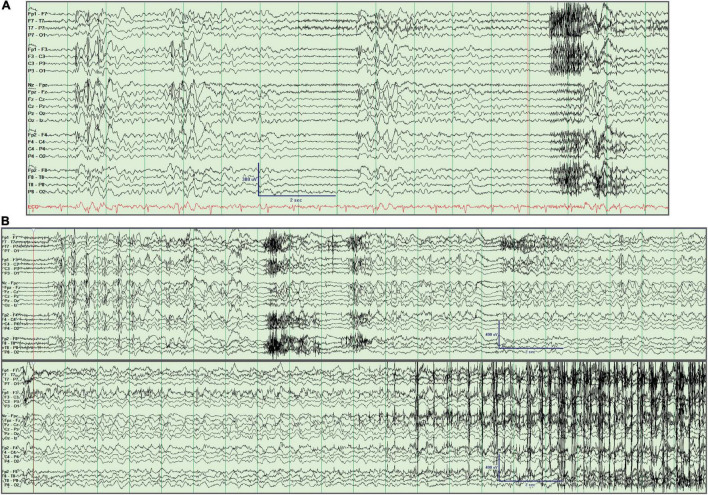
Interictal and ictal EEG recording. **(A)** Generalized spike/polyspike and wave discharges. **(B)** Generalized onset clonic-tonic-clonic seizure. The seizure starts 2 s into the tracing and is seen in the continuation of the EEG in the lower panel, with evident clonic myogenic artifact near the end of the recording. This activity was preceded by recurrent bursts of generalized spike/polyspike and wave discharges. Longitudinal bipolar montage left over right.

Her brain MRI was unremarkable. Epilepsy monitoring unit (EMU) video EEG recorded frequent generalized 4–5 Hz atypical spike-wave and polyspike and wave interictal epileptiform discharges (IEDs) which intermittently became nearly continuous. One generalized onset clonic-tonic-clonic seizure was recorded with clonic activity of both arms, bilateral eyelid twitching, and late left head turning and left arm extension followed by generalized tonic-clonic activity (Figure 1). Another clinical episode occurred in the setting of hyperventilation in which she felt dizzy and had difficulty speaking and reading without EEG correlate, which was deemed to represent a PNES. No absence seizures were recorded.

After optimization of her ASMs, including discontinuation of oxcarbazepine, addition of brivaracetam 100 mg twice daily, Clonazepam 0.5 mg twice daily, VNS optimization, and initiation of the modified ketogenic diet, she continued to have frequent seizures. Her case was presented for review at our multidisciplinary epilepsy conference for consideration of CM stimulation given ongoing monthly convulsive seizures, and concern for seizure related morbidity or mortality [including sudden unexpected death in epilepsy (SUDEP)]. Off-label use of FDA-approved devices for CM DBS and responsive neurostimulation (RNS) were discussed at the meeting, including evidence for safety, feasibility, and possible efficacy. A consensus was reached that CM DBS or CM RNS would be reasonable treatment approaches. After careful discussion with the patient and review of the potential risks and benefits, the shared decision was to proceed with CM DBS as a palliative treatment. In the 3 months preceding DBS placement she had 4 convulsive seizures and 2 episodes of convulsive status epilepticus and intermittent episodes of staring that occurred at least weekly.

**FIGURE 2:**
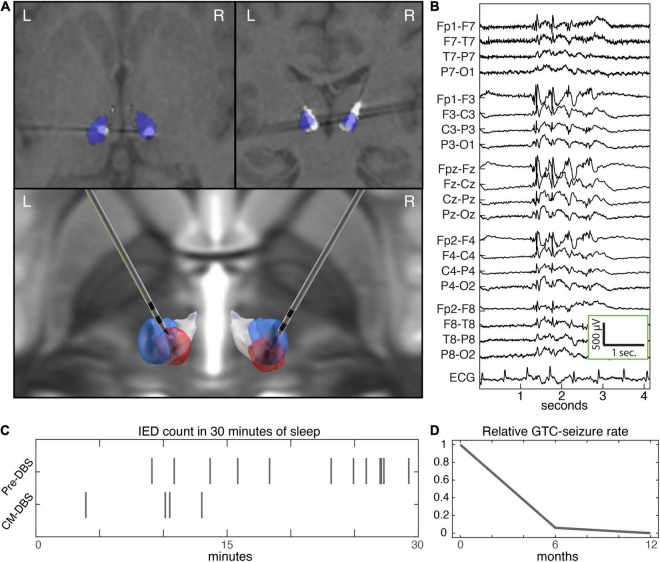
Centromedian DBS and epilepsy biomarkers. **(A)** Axial (top left) and coronal (top right) sections of post-operative CT coregistered to pre-operative T1 MP-RAGE MRI, and rendered implantation (bottom). The CM and Pf nuclei are colored blue and white, respectively. Neurological orientation (left hemisphere is left). The estimated VTA (red) based on stimulation parameters at last follow-up (VTA modeling with Lead-DBS SimBio/FieldTrip) (Horn and Kühn, 2015). **(B)** Interictal generalized spike and wave discharges. **(C)** Raster plot of epileptiform discharges during initial 30 min of stage N2 sleep at baseline and with CM DBS. **(D)** Seizure rate at baseline over the 6-months preceding DBS, and during 1 year of follow up with CM DBS. CM, centromedian; DBS, deep brain stimulation; MP-RAGE, magnetization-prepared rapid gradient echo; Pf, parafascicular; VTA, volume of tissue activated.

A presurgical localizing stereotactic MRI was performed followed by stereotactic placement using a rigid Leksell frame. Indirect targeting of the CM-Pf complex relied on anterior commissure-posterior commissure (AC-PC) based offsets and Montreal Neurological Institute (MNI) template space Morel atlas structures (Morel et al., 1997) warped into patient space as previously described in a trial of CM DBS for epilepsy (LGS) (Warren et al., 2020). Final AC-PC offsets were 7 mm lateral from midline, 12 mm posterior from the mid-commissural point, and 1 mm superior to the AC-PC plane. Four contact depth electrodes with 1.5 mm spacing (Medtronic 3387) were implanted bilaterally under stereotactic guidance and connected to a Medtronic PC-Activa™ implantable pulse generator. Postoperative head CT coregistered to preoperative MRI demonstrated good positioning (Figure 2), using the Lead-DBS imaging package (Horn and Kühn, 2015)^1^ and Morel atlas structures (Morel et al., 1997; Morrell and System in Epilepsy Study Group, 2011) demonstrated well positioned leads (Figure 2).

Electrode positions were planned using indirect AC-PC offsets and volumetric thalamus atlas structures warped into the patient’s imaging space (Figure 2). Post-operative imaging confirmed well positioned electrodes. During the post-implantation hospital stay our patient completed a monopolar review and paresthesias were noted with stimulation of the more superficial 2 electrode contacts, likely reflecting involvement of the ventralis caudalis (Vc) nucleus in the volume of tissue activated (VTA) (McCabe et al., 2021).

Given the patient’s history of frequent GTC seizures, the DBS was started at low settings postoperative day one: referential single contact cathodal stimulation through the deepest contact, 60 hertz (Hz), 90 microsecond (μsec) pulse width, and 1 volt (V) amplitude, delivered to the deep electrode contact bilaterally. The amplitude was increased at home to 2V bilaterally after 10 days using the patient programmer, and at the 3 month follow up visit the same stimulation program was increased from 2 to 3 V.

Two months after this session, the patient’s medications were reduced by her local provider due to improved seizure control because of side effects and a short time later she had a GTC seizure lasting 3 min. Once medications were resumed, she did not have any GTCs for the next 3 months. Later, she suffered from an unrelated illness requiring hospitalization during which medications were inadvertently reduced, resulting in a cluster of GTC seizures. After recovering from her illness and restarting her ASMs at the prescribed doses, she did not have any more GTC seizures. Over the next 6 months the program voltage was gradually increased to 4.5 V (therapy current: left lead 4.9 mA, right lead 4.3 mA) (Figure 2). An overnight ambulatory EEG performed 1 year after her implant recorded rare generalized IEDs seen only during sleep and no seizures were recorded. There was an apparent reduction in IED rate at follow up with CM DBS (Figure 2).

At her 12 month follow up visit she did not have any more GTC seizures. She continued to report absence seizures as before; the contribution from PNES, as recorded in the EMU, is unknown. The patient and her family were very pleased with the outcome from DBS-CM and endorsed improvement in her quality of life. She made lifestyle changes which included decreasing alcohol intake. Her ASM regimen remained Lamotrigine 250 mg twice daily, Zonisamide 400 mg twice daily Brivaracetam 100 mg twice daily, Clonazepam 0.5 mg twice daily at her last follow-up. At her 12 month follow-up she reported that she was 6 weeks pregnant and during her pregnancy she had not had any GTCs.

## Discussion

This report describes the case of the first cognitively normal individual with drug resistant GGE to be treated with CM DBS at our institution, and her case is notable for a marked reduction in GTC seizures. Our patient has been free from GTC seizures for the 6 months preceding last follow up, and the two GTC seizure days since initiation of CM DBS occurred in the setting of medication withdrawal and illness. The monthly seizure frequency reduction of GTCs in the first 6 months post implant was 94% and in the 12 months following stimulation was 97%. She did not report any change in the burden of absence seizures, however some questions remain regarding the nature of these events since no absence seizures were recorded over 5 days of video EEG monitoring with medication withdrawal. This case contributes to the small existing body of work that suggests CM DBS may be an effective therapy for cognitively normal patients with drug resistant GGE, or IGE-like epilepsy (Velasco et al., 2006; Kwan et al., 2011; Scheffer et al., 2017).

The CM nucleus is a part of the caudal group of the intralaminar thalamic nuclei. CM is a part of a diffuse network with connections to the brainstem ascending reticular system, basal ganglia, direct cortical projections, and interconnections to other thalamic nuclei (Velasco et al., 1989, 1993, 2006, 2021; Valentín et al., 2012; Cukiert et al., 2020; Alcala-Zermeno et al., 2021; Torres Diaz et al., 2021). Studies have shown that CM stimulation can desynchronize thalamocortical loops and modulate circuits responsible for the hypersynchronous activity that plays a role in seizure onset and propagation (Alcala-Zermeno et al., 2021), which may underlie the efficacy of CM DBS in seizures involving loss of awareness (Velasco et al., 1989, 1993, 2006, 2021; Fisher et al., 1992; Valentín et al., 2012; Cukiert et al., 2020; Kokkinos et al., 2020; Alcala-Zermeno et al., 2021; Torres Diaz et al., 2021).

At present there have been 3 RCTs (Fisher et al., 1992; Velasco et al., 2000; Dalic et al., 2022) and 9 non-controlled studies (Velasco et al., 1987, 1989, 1993, 2002, 2006; Velasco M. et al., 2001; Chkhenkeli et al., 2004; Kim et al., 2013; Son et al., 2016; Cukiert et al., 2020; Alcala-Zermeno et al., 2021) of CM DBS in largely heterogenous cohorts of patients with drug resistant DEE, focal epilepsy, as well as small numbers of cognitively normal individuals with GGE (Cukiert et al., 2009; Li and Cook, 2018; Yan et al., 2018; Torres Diaz et al., 2021; Velasco et al., 2021). The evidence from these studies is mixed, but suggests that CM DBS is safe and can be an effective therapy for LGS or DEE (Fisher, 2013; Yan et al., 2018; Velasco et al., 2021). The RCT by Fisher et al failed to meet its primary endpoint, however there was 50% responder rate at the end of the open label follow up (Fisher et al., 1992). The RCT by Velasco et al. (2000) did not achieve a statistically significant seizure reduction in the 3 month blinded phase, however, they did report a significant reduction in GTCs and absence seizures and a lack of impact on focal onset seizures. Both studies were comprised of heterogenous cohorts including individuals with drug resistant focal or generalized epilepsy. The recent RCT of CM DBS for LGS did not meet its primary endpoint based on patient reported diary seizures, however there was a significant reduction in electrographically defined seizures on 24-h ambulatory EEG at the end of the blinded phase (Dalic et al., 2022). All three RCTs used duty cycle DBS (1 min on, 4–5 min off), in contrast to some other work (Cukiert et al., 2009; Valentín et al., 2013). Most studies were with small sample sizes with the largest study involving 49 patients (Velasco et al., 2002). Most of the studies also noted an implant effect with seizure reduction without stimulation which may have impacted the statistical significance of the RCTs. Of these, only two studies clearly included cognitively normal patients with GGE or IGE (Valentín et al., 2013; Cukiert et al., 2020). In the study by Valentín et al. (2013) all 4 patients with IGE showed a greater than 50% improvement in seizure frequency at 12 months follow up. In that study, stimulation parameters were continuous stimulation at 60 Hz, pulse width of 90 μs, and amplitude up to 5 V, comparable to the parameters used in our subject. In the study by Cukiert et al. (2009), 2 patients with GGE with previous corpus callosotomy showed a 70% and 80% improvement in seizure frequencies at 12 months. In this study, the stimulation parameters were continuous stimulation at 130 Hz, 300 μs and amplitude of 2 V.

Our patient did not report any side effects with gradually increased monopolar stimulation amplitudes delivered to the deep contact positioned in the CM-Pf complex bilaterally. Paresthesias are a known side effect with CM stimulation however stimulation was well tolerated throughout the follow up period and did not limit therapy in our patient. Our patient had a relatively medial target location compared to prior work, providing greater separation from the Vc nucleus while maintaining CM-VTA correspondence.

The control of her GTC seizures is particularly relevant given the multiple episodes of status epilepticus and hospital admissions with injuries in the year prior to her DBS implant. This reduction in GTC seizures reduces SUDEP risk (McCabe et al., 2021), hospitalization and status epilepticus rates, seizure-related morbidity, and improves quality of life.

It is important to note the patient made lifestyle changes that could influence seizure risk by reducing alcohol intake before her 6 months follow up visit. It is unclear if this was due to a reduced need for alcohol to control anxiety or for other reasons. She was 6 weeks pregnant at her 12 month follow up visit, and GTC seizure control was sustained at that time. The only breakthrough GTC seizures that she experienced were in the setting of medication changes or during a hospitalization for unrelated illness, but she had overall better medication compliance over time.

This is a single case report of a young, cognitively normal woman with drug resistant GGE who had marked reduction in the frequency of GTC seizures following treatment with CM DBS, achieving 6 months of GTC seizure freedom at last follow up 12 months after surgery. Current evidence in the literature for CM DBS for cognitively normal individuals with drug resistant GGE (IGE-like epilepsy) is limited to a total of 7 subjects, including our patient, all of whom are reported to be responders. This case highlights the potential role of CM as a target for cognitively normal individuals with GGE. This work underscores the need for future prospective studies of CM DBS for the underserved group of individuals with drug resistant GGE. Future efforts will benefit from reliable reporting of epilepsy classification, electrode targeting, and DBS stimulation parameters. Ultimately, prospective clinical trials are warranted to evaluate this potentially effective therapy for cognitively normal individuals with drug resistant GGE.

## Data Availability Statement

The original contributions presented in the study are included in the article/supplementary material, further inquiries can be directed to the corresponding authors.

## Ethics Statement

This single patient retrospective chart review is exempt from IRB review: “The Mayo Clinic Institutional Review Board deemed this retrospective case review to be exempt from formal review in accordance with the Code of Federal Regulations, 45 CFR 46.102.” Written informed consent for participation was not required for this study in accordance with the national legislation and the institutional requirements.

## Author Contributions

SA and NG contributed to conception and design of the study and wrote the manuscript. DB and JV followed the subject. All authors contributed to the revision of the manuscript and approved submission of the current version.

## Conflict of Interest

GW, BL, and JV declared intellectual property licensed to Cadence Neuroscience. BL, NG, JV, and GW were investigators for the Medtronic Deep Brain Stimulation Therapy for Epilepsy Post-Approval Study. BL was an investigator for the NeuroPace RNS System Responsive Stimulation for Adolescents with Epilepsy (RESPONSE) study and Neuroelectrics tDCS for Patients with Epilepsy Study. As part of National Institutes of Health grants UH2&3-NS95495 and UG3NS112826-01, Medtronic provided medical devices free of charge (GW, BL, JV, and NG). The remaining authors declare that the research was conducted in the absence of any commercial or financial relationships that could be construed as a potential conflict of interest.

## Publisher’s Note

All claims expressed in this article are solely those of the authors and do not necessarily represent those of their affiliated organizations, or those of the publisher, the editors and the reviewers. Any product that may be evaluated in this article, or claim that may be made by its manufacturer, is not guaranteed or endorsed by the publisher.

## Footnotes

1
https://www.lead-dbs.org
